# Cytotoxicity of Mycotoxins and Their Combinations on Different Cell Lines: A Review

**DOI:** 10.3390/toxins14040244

**Published:** 2022-03-30

**Authors:** Paweł Skrzydlewski, Magdalena Twarużek, Jan Grajewski

**Affiliations:** Department of Physiology and Toxicology, Faculty of Biological Sciences, Kazimierz Wielki University, 85-064 Bydgoszcz, Poland; paskrzy@ukw.edu.pl (P.S.); jangra@ukw.edu.pl (J.G.)

**Keywords:** cytotoxicity, mycotoxins, cell line, apoptosis, MTT

## Abstract

Mycotoxins are secondary metabolites of molds and mainly produced by species of the genera *Aspergillus*, *Penicillium* and *Fusarium*. They can be synthesized on the field, during harvest as well as during storage. They are fairly stable compounds and difficult to remove. Among several hundreds of mycotoxins, according to the WHO, ochratoxin A, aflatoxins, zearalenone, deoxynivalenol, patulin, fumonisins as well as T-2 and HT-2 toxins deserve special attention. Cytotoxicity is one of the most important adverse properties of mycotoxins and is generally assessed via the MTT assay, the neutral red assay, the LDH assay, the CCK-8 assay and the ATP test in different cell lines. The apoptotic cell ratio is mainly assessed via flow cytometry. Aside from the assessment of the toxicity of individual mycotoxins, it is important to determine the cytotoxicity of mycotoxin combinations. Such combinations often exhibit stronger cytotoxicity than individual mycotoxins. The cytotoxicity of different mycotoxins often depends on the cell line used in the experiment and is frequently time- and dose-dependent. A major drawback of assessing mycotoxin cytotoxicity in cell lines is the lack of interaction typical for complex organisms (for example, immune responses).

## 1. Introduction

Mycotoxins are toxic compounds mainly produced by species of the genera *Aspergillus*, *Fusarium* and *Penicillium*. They can be detected in numerous foodstuffs, including nuts, spices, cereals and fruits, both pre- and post-harvest. Mold growth and, consequently, mycotoxin production can be affected by numerous factors such as weather conditions. The thermal stability and ability of mycotoxins to withstand food processing is one of the main reasons for concerns. Out of several hundreds of mycotoxins most commonly occurring and threatening human and animal health, we highlight patulin, aflatoxin, ochratoxin A, fumonisin, deoxynivalenol, T-2 and HT-2 toxins, zearalenone, citrinin and enniatin [1].

### 1.1. Patulin

Patulin is a mycotoxin produced by several species of the genera *Aspergillus* and *Penicillium*, mainly by *Penicillium expansum* in rotten fruits and fruit juices. The temperatures at which *P. expansum* can produce patulin range from 0 to 24 °C [2].

### 1.2. Aflatoxins

Aflatoxins are produced by *Aspergillus* species, mainly by *Aspergillus parasiticus* and *Aspergillus flavus*. They can be divided into Aflatoxin B1, B2, G1 and G2. These mycotoxins can be produced during storage as well as on the field, typically in warmer and more humid climates. Intoxication by aflatoxins can lead to immune system suppression, child development impairment, cancer or, in severe cases, death [3].

### 1.3. Ochratoxin A

Ochratoxin is a mycotoxin that occurs during storage under inadequate conditions. This mycotoxin is mainly produced by *Penicillium verrucosum, Aspergillus ochraceus* and *Aspergillus carbonarius* [4].

### 1.4. Fumonisins

Fumonisins most commonly occur in dry and warm regions and often contaminate maize. They are mainly produced by *Fusarium* species, including *Fusarium proliferatum* and *Fusarium verticilioides*. Among several fumonisins, fumonisin B1 is the most common one [5].

### 1.5. Deoxynivalenol

This mycotoxin is one of the trichothecenes, produced mainly by *Fusarium culmorum, Fusarium graminearum* and *Fusarium nivale*. It is generated during growth and harvest periods in relatively humid and cool weather conditions [6].

### 1.6. T-2, HT-2 Toxins

The T-2 toxin and its derivative HT-2 toxin are further examples of trichothecenes produced mainly by *Fusarium* species. High levels of these toxins are found mostly in developing countries as they require high temperatures and relatively high humidity (prolonged rains during harvest, monsoons, flash floods) to produce mycotoxins [7].

### 1.7. Zearalenone

Zearalenone is largely produced during the growth period in moderate climates with relatively high humidity. The main producers are *Fusarium moniliforme*, *F. culmorum*, *F. graminearum*, *F. oxysporum*, *F. sporotrichides* and *F. crookwellence* [8,9].

### 1.8. Citrinin

Citrinin is a mycotoxin produced by several species of *Penicillium*, *Aspergillus* and *Monascus*. It most frequently occurs in rice (where it is responsible for yellow rice disease in Japan), cereals, fruits and cheese [10].

### 1.9. Enniatin

Enniatins are secondary metabolites mainly produced by the genera *Fusarium* and comprised of Enniatin A, Enniatin A1, Enniatin B and Enniatin B1. They can be found in nuts, spices, fruits, cocoa, coffee, several grains as well as their products. Enniatins are considered as emerging mycotoxins [11].

### 1.10. Cytotoxicity

Cytotoxicity is defined as the ability to harm living cells, causing, among others, protein synthesis disruption or weakening of the cell membrane, ultimately leading to cell death (both necrotic and apoptotic) [12,13]. Cytotoxicity can be measured by various methods, including the 3-(4,5-dimethylthiazol-2-yl)-2,5-diphenyltetrazolium bromide (MTT) test, CCK-8 test, the ATP test, the lactate dehydrogenase (LDH) test, the AlamarBlue™ assay and the neutral red uptake test. [14]. Of these, the MTT assay relies on the reduction in 3-(4,5-dimethylthiazol-2-yl)-2,5-diphenyltetrazolium bromide by mitochondrial dehydrogenase to purple formazan crystals. After dissolution in DMSO, formazan is measured spectrophotometrically (~550 nm).

Since only living cells can reduce MTT to formazan, the amount of formazan is used to assess the number of living cells [15]. The CCK-8 test is a test similar to the MTT. In this test, a highly water-soluble reagent, WST-8 [2-(2-methoxy-4-nitrophenyl)-3-(4-nitrophen-yl)-5-(2,4disulfophenyl)-2H-tetrazolium, monosodium salt], is reduced by dehydrogenase in living cells to formazan (a colored product). The amount of formazan is directly correlated to the number of living cells [16]. In the ATP test, the amount of ATP is measured. In this approach, MgATP2- converts luciferin, obtained from fireflies, into a form which can be oxidized catalytically by luciferase in a chemiluminescent reaction. Light intensity (~562 nm) is directly correlated to the amount of ATP [17]. The LDH test measures lactate dehydrogenase activity in culture media. Since LDH is a cytoplasmic enzyme, it is rapidly released into culture media during necrosis or apoptosis (cell membrane damage). In this test, tetrazolium salt (yellow color) is converted by NADH to formazan dye (red color), which can be measured spectrophotometrically (~492 nm). The amount of formazan dye is directly correlated to the amount of LDH in culture media (and to the number of damaged cells) [18].

The neutral red uptake (NR, NRU) assay relies on the ability of living cells to absorb neutral red dye. The dye penetrates cells via non-ionic diffusion and is accumulated in the lysosomes. After washing, the dye is extracted from cells using acidified ethanol and measured spectrophotometrically. This assay relies on the capability of cells to retain a pH gradient which allows the dye to penetrate the cell membrane and retain in the lysosomes. Since only living cells have this ability, the amount of measured dye is directly correlated with the number of living cells [19]. The AlamarBlue™ assay is a cell viability assay using a non-toxic, weakly fluorescent blue dye, resazurin. In healthy, live cells, resazurin is reduced to a pink, highly fluorescent dye, resorufin. The intensity of fluorescence is proportional to the number of respiring, living cells. This assay is used as an alternative to other tests, for example, the MTT [20].

### 1.11. Cell Lines Used for Cytotoxicity Assessment

Various different cell lines are used for cytotoxicity assessment. The cell lines used in the reviewed literature are summarized in Table 1.

## 2. Results

All reviewed articles are summarized in Supplementary Table S1.

### 2.1. Single Mycotoxins

#### 2.1.1. Patulin

In the case of patulin, MTT was the most frequently used test, and HepG-2 was the most frequently used cell line. Results showed a decrease in cells’ viability, as well as ROS formation increase, cell cycle arrest and p53 gene expression. IC50 values for patulin range from µM to mM, depending on experiment conditions, time of incubation and cell line used (full results are presented in Table 2).

#### 2.1.2. Aflatoxins

In case of aflatoxins, MTT was the most frequently used test, followed by LDH release and NRU, while HepG-2 and Caco-2 were the most frequently used cell lines. Results showed a decrease in cells’ viability, as well as increase in the apoptotic cell ratio, increased ROS production and cell cycle arrest. IC50 values for aflatoxins range from nM to µM, depending on experiment conditions, time of incubation and cell line used (full results are presented in Table 3).

#### 2.1.3. Ochratoxin A

In case of ochratoxin A, MTT was the most frequently used test, followed by CCK-8, while HepG-2 and Caco-2 were the most frequently used cell lines. Results showed a decrease in cells’ viability, as well as an increase in the apoptotic cell ratio, increased ROS production and cell cycle arrest. IC50 values for ochratoxin A range from 1.86 µM to >200 µM, depending on experiment conditions, time of incubation and cell line used (full results are presented in Table 4).

#### 2.1.4. Fumonisins

In the case of fumonisins, MTT was the most frequently used test, followed by LDH release, while HepG-2 and Caco-2 were the most frequently used cell lines. Results showed that, even at high concentration, fumonisins are significantly less cytotoxic than other tested mycotoxins (full results are presented in Table 5).

#### 2.1.5. Deoxynivalenol

In the case of deoxynivalenol, MTT was the most frequently used test, followed by NRU, while HepG-2 was the most frequently used cell line, followed by Caco-2. Results showed a decrease in cells’ viability, as well as an increase in the apoptotic cell ratio and increased ROS production. IC50 values for deoxynivalenol range from nM to µM, depending on experiment conditions, time of incubation and cell line used (full results are presented in Table 6).

#### 2.1.6. T-2 and HT-2 Toxins

In the case of T-2 and HT-2 toxins, MTT was the most frequently used test, followed by LDH release and NRU, while HepG-2 was the most frequently used cell lines followed by Vero and Leyding cells. Results showed a decrease in cells’ viability, as well as an increase in the apoptotic cell ratio, increased ROS production. It was also shown that a strong cytotoxic effect occurs even at low concentrations (full results are presented in Table 7).

#### 2.1.7. Zearalenone

In the case of zearalenone, MTT was the most frequently used test, followed by NRU, while HepG-2 was the most frequently used cell line, followed by Caco-2 and CHO-K1. Results showed a decrease in cells’ viability, as well as an increase in the apoptotic cell ratio, inhibition in protein and DNA synthesis and increased ROS production. IC50 values for zearalenone range from ~10 µM to >100 µM, depending on experiment conditions, time of incubation and cell line used (full results are presented in Table 8).

#### 2.1.8. Citrinin

In the case of citrinin, MTT was the most frequently used test. Results showed a decrease in cells’ viability, as well as an increase in the apoptotic cell ratio, increased ROS production and cell cycle arrest. IC50 values for citrinin varies depending on experiment conditions, time of incubation and cell line used (full results are presented in Table 9).

#### 2.1.9. Enniatins

In the case of enniatins, MTT was the most frequently used test, followed by AlamarBlue™, while Caco-2 was the most frequently used cell line, followed by HepG-2. Results showed a decrease in cells’ viability, as well as an increase in the apoptotic cell ratio and cell cycle arrest. IC50 values for enniatins vary, depending on experiment conditions, time of incubation, cell line used and specific enniatin tested (full results are presented in Table 10).

### 2.2. Multiple Mycotoxins

In the case of mycotoxins’ mixtures, their combined effect can be calculated using the combination index (CI), a method derived from the median effect principle [98,99]. The *CI* value is calculated from the general equation:

(1) CIxn=∑j=1nDj(Dx)j

where:

^n^(*CI*)*_x_* is combination index for n components at *x*% of cell proliferation inhibition
(*D*)*_j_* is the dose of n mycotoxins that causes *x*% cell proliferation inhibition in the combination
(*D_x_*)*_j_* is the dose of each n mycotoxin individually that causes *x*% cell proliferation inhibition

*CI* < 0.9, *CI* = 0.9–1.1, *CI* > 1.1 generally indicate synergistic, additive and antagonistic effects, respectively. Additionally, when the *CI* indicates a synergistic effect, dose reduction indices (*DRI*) can be calculated. *DRI* indicate a fold of reduction of dose of each component at a given effect level compared to a dose of each component individually and can be calculated from equation [100]:

(2) CIxn=∑j=1nDj(Dm)j=∑j=1n1DRIj and  DRIj=Dj(Dm)j

where:

*DRI* is dose reduction indices
*D_m_* is median effect dose

#### 2.2.1. Combination of Two Mycotoxins

In the case of combinations of two mycotoxins, MTT was the most frequently used test, while HepG-2 was the most frequently used cell line. Results showed that cytotoxicity of binary combinations strongly depend on components (while some mixtures exhibit synergistic effects, others exhibit additive ore antagonistic effects). Moreover, a concentration of individual components seems to have an effect on mixture cytotoxicity (full results are presented in Table 11).

#### 2.2.2. Combination of Three Mycotoxins

In the case of combinations of three mycotoxins, MTT was the most frequently used test, while HepG-2 and Caco-2 were the most frequently used cell line. Results showed that cytotoxicity of tertiary combinations strongly depend on components (while some mixtures exhibit synergistic effects, others exhibit additive ore antagonistic effects). Moreover, a concentration of individual components seems to have effect on mixture cytotoxicity (full results are presented in Table 12).

#### 2.2.3. Combination of Four Mycotoxins

Prosperini et al. [91] tested the cytotoxicity of mixtures of four enniatins against Caco-2 cells. The MTT assay showed that this mixture reduced cell viability in a dose-dependent manner by approximately 40%.

## 3. Conclusions

In conclusion, among various negative properties, cytotoxicity is one of the most important one of mycotoxins. To assess this cytotoxicity, the most frequently chosen assay is MTT, followed by the neutral red assay, the CKK-8 assay, the AlamarBlue™ assay and the LDH cytotoxicity assay. For apoptotic cell ratio assessment, flow cytometry is most frequently being used. As shown in this review, most mycotoxins exhibit cytotoxic properties in a dose- and time-dependent manner; however, the concentration of those mycotoxins range from nM to µM. The cell line chosen in the mycotoxin cytotoxicity study is also important because different cell lines react differently to certain mycotoxins. Under natural conditions, several mycotoxins often co-occur, making it important to assess the cytotoxic effects of different combinations of mycotoxins. Based on the findings of this review, such combinations often exhibit different levels of cytotoxicity compared to the individually applied mycotoxins, with a stronger cytotoxicity. A major drawback of the cytotoxicity assessment in cell lines is the lack of interaction with different cell types and mechanisms naturally occurring in complex organisms (for example, immunology responses). This problem could be partially solved by using primary cell lines instead of continuous cell lines (since they retain many characteristics of cells in vivo; this however generates a problem with reproducibility of the results due to, e.g., viral or bacterial contamination) or by using 3D printed models of tissues to emulate the natural environment better.

## Figures and Tables

**Table 1:** Summary of cell lines used in reviewed literature.

Cell Line	Description
HepG-2	Human liver cancer cell line derived from a liver hepatocellular carcinoma of a 15-year-old Caucasian male
CHO-K1	Cell line derived as a subclone from the parental CHO cell line initiated from a biopsy of an ovary of an adult Chinese hamster
HEK293	Cell line derived from human embryonic kidney cells grown in tissue culture
BME	Bovine mammalian epithelial cell line
Caco-2	Immortalized cell line of human colorectal adenocarcinoma cells; can spontaneously differentiate into a heterogeneous mixture of intestinal epithelial cells
SK-N-SH	Neuroblastoma cell line that displays epithelial morphology and grows in adherent culture
BRL 3A	Originated from Buffalo Rat liver, isolated by primary cloning. Serum-free medium conditioned by the cells produces a family of polypeptides termed multiplication stimulating activity (MSA)
HK-2	Immortalized proximal tubule epithelial cell line from normal adult human kidney
Het-1A	Derived from human esophageal autopsy tissue by transfection with plasmid pRSV-T consisting of the RSV-LTR promoter and the sequence encoding the simian virus 40 large T-antigen
GES-1	Human gastric epithelial cells
IPEC-12	Intestinal porcine enterocytes isolated from the jejunum of a neonatal unsuckled piglet
Jurkat T	Immortalized line of human T lymphocyte cells that are used (among others) in research involving the susceptibility of cancers to drugs and radiation
TM3 Leydig	Originated from primary cultures of Leydig cells with similar characteristics as the original cell line
SerW3	Sertoli cell line derived from rat, immortalized by the T-antigen of the Simian virus
Vero	Continuous and aneuploidy cell line isolated from kidney epithelial cells extracted from an African green monkey
V79	Fibroblast cell line originally derived from the lung of a normal Chinese hamster (male). The G1 phase in these cells is either absent or very short
C5-O	Mice keratinocyte cell line
H9c2	Immortalized cells with a cardiac phenotype widely used for the analysis of cardiac IR injury and ischemic preconditioning
PK-15	Porcine kidney epithelial cell line
MAC-T	Bovine mammalian epithelial cells, derived from primary bovine mammary alveolar cells, transfected with SV-40 large T-antigen
BRL	Rat liver cell line
HTC116	Human colon cancer cell line, used mainly for drug and therapeutic research
TM4	Mouse BALB/c testis Sertoli cell
RPTEC	Derived from primary human renal proximal tubular epithelial cells
NHLF	Normal human lung fibroblasts
BF-2	Cells derived from 1-year-old fingerlings of *Lepomis macrochirus*
BEAS-2B	Human non-tumorigenic lung epithelial cell line
PBM	Peripheral blood mononuclear cells
RTH-149	Rainbow trout hepatoma cells
PLHC-1	*Poeciliopsis lucida* hepatocellular carcinoma, derived from adult female of *Poeciliopsis lucida*
H4IIE	Rat hepatoma cell line
K562	Lymphoblasts isolated from the bone marrow of a 53-year-old chronic myelogenous leukemia patient
PBL	Peripheral blood lymphocytes
PBG	Peripheral blood granulocytes
SK	Swine kidney cells
HeLa	Immortal cell line derived from cervical cancer. Oldest human cell line
MDCK	Madin-Darby canine kidney cells
MDBK	Madin-Darby bovine kidney cells
RTGill-W1	Rainbow trout gill cell line derived from fragments of 15-month-old rainbow trout
BEL	Bovine embryonic lung cells
RTL-W1	Rainbow trout liver cell line
RT EQ clone 8	Epithelial gonadal cell line derived from rainbow trout
CCB	Carp brain cells
SHK-1	Salmon head kidney cells
HKC	Human renal proximal cell line
PKC	Primary porcine kidney cells
LLC-PK1	Pig kidney epithelial cell derived from a male pig
PFBK	Primary fetal bovine kidney cells
MRC-5	Fibroblast-like fetal lung cell line
HT-29	Human colorectal adenocarcinoma cell line
IHKE	Immortalized human proximal tubule cells
A549	Adenocarcinomic human alveolar basal epithelial cells

**Table 2:** Cytotoxicity of patulin tested on different cell lines.

Type of Test	Cell Line	Results	Reference
MTT	HepG-2	Dose- and time- dependent decrease in cell viability from 92% to 13% after 24 h of incubation and to 2% after 48 and 72 h	[21]
MTT	CHO-K1	Patulin decreased cell proliferation from 45 to 16% in all tested time periods (24, 48 and 72 h) at concentrations 3.125 and 6.25 µM	[22]
MTT, NRU	CHO-K1	Patulin decreased cell viability in a dose-dependent manner, with IC50 values of 4.4 µM (NRU) and 0.69 µM (MTT) after 24 h of incubation. The ROS concentration was also significantly increased after incubation with patulin	[23]
MTT	HEK293	Treatment with patulin at concentrations of 2.5, 5, 7.5, 10 and 15 μM resulted in dose-dependent viability decreases by 8, 18, 31.0, 42 and 63%, respectively	[24]
MTT	HepG-2	Marked drop in cell viability after 24 h of incubation with patulin at concentrations ranging from 5 to 100 µM in a dose-dependent manner. Patulin also increased ROS formation as well as p53 gene expression	[25]
MTT, LDH release, Hoechst 33,258 dye	H9c2	IC50 of patulin at 25 µM. Patulin also increased LDH release and increased the apoptotic cell ratio	[26]
CCK-8	HepG-2	Dose- and time-dependent reduction in cells’ viability. Incubation with patulin for 10 at concentrations from 2.5 to 15 µM resulted in a reduction in cell viability from 91.46 to 53.16%, respectively; the IC50 after 10 h of incubation was 15.85 µM. Patulin also increased lipid peroxidation and decreased the levels of antioxidant, stress-related enzymes	[27]
MTT	Caco-2	Dose-dependent decrease in cell viability after 24 h of incubation with patulin at concentrations ≥ 25 µM. The IC50 for patulin was calculated at 15.95 µM	[28]
MTT	HepG-2	Decreased cell viability in a dose-dependent manner; the LC50 was 7.2 mM after 24 h of incubation. Adding autophagosome formation inhibitor/promotor and ROS inhibitor proved that reduction in cell viability was due to autophagy	[29]
MTT, flow cytometry	HCT116	Decreased cells’ viability by inducing apoptosis via G2/M arrest. The MTT assay showed a dose-dependent decrease in cell viability, whereas flow cytometry showed an increase in number of apoptotic and G2/M cells	[30]
MTT	SK	In this study, cytotoxicity was defined as a decrease in cell viability by 20%. This effect was achieved after 24 h of incubation with patulin at a concentration of 0.4 µg/mL	[31]

**Table 3:** Cytotoxicity of aflatoxins tested on different cell lines.

Type of Test	Cell Line	Results	Reference
CCK-8	BME	Aflatoxins AFB1 and AFM1 exhibited cytotoxic properties in a dose- and time-dependent manner at various concentrations after 24 and 48 h of incubation. They also induced apoptosis and increased the ratio of cells in the G1 and G2 phases.	[32]
MTT, LDH release	Caco-2, Hep-G2, SK-N-SH	Both aflatoxins AFB1 and AFM1 decreased the viability of cells by damaging the cell membrane.	[33]
MTT	Caco-2	Aflatoxin AFM1 inhibited cell viability in a dose- and time-dependent manner after 24, 48 and 72 h of incubation.	[34]
MTT, LDH release	BRL 3A	AFB1 reduced cell viability in a dose- and time-dependent manner. AFB1 also increased LDH activity, apoptotic cell ratio and ROS production.	[35]
MTT, NRU	Caco-2, Raw264.7, MDBK	AFB1 exhibited cytotoxic properties against MDBK, reducing cell viability by 21% after 48 h of incubation with AFB1 at a concentration of 3.8 µg/mL. No significant decrease in cell viability was observed in Raw264.7 and Caco-2 cell lines.	[36]
Cell Proliferation Reagent WST-1	BME-UV1	Aflatoxin B1 is cytotoxic against the BME-UV1 cell line in a dose- and time-dependent manner, with LC50 values of 687 and 180 nM after 24 and 48 h, respectively.	[37]
MTT, NRU	BME-UV1	Aflatoxin caused a decrease in cell viability in a dose- and time-dependent manner. NRU tests showed that after 72 h of incubation, cell viability was decreased by more than 70% in all concentrations tested. The MTT test also showed a significant decrease in cell viability in all concentrations tested after 24 h of incubation.	[38]
MTT	Caco-2	Aflatoxin B1 and M1 exhibited cytotoxic properties against the Caco-2 cell line. The MTT assay showed a significant dose- and time-dependent decrease in cell viability, both differentiated and undifferentiated cells, when treated with mycotoxins. It was shown that aflatoxin B1 is more cytotoxic than aflatoxin M1.	[39]
MTT, LDH release	PK-15	Aflatoxin B1 exhibited dose- and time-dependent cytotoxic properties. The MTT test showed that after 48 h of incubation, the IC50 for aflatoxin B1 was 38.8 µM. Regarding the LDH release, an AFB1 in concentration of 24.9 µM caused an increase in LDH release by 30% after 24 h of incubation.	[40]
Cell Proliferation ELISA BrdU Kit, Flow cytometry	MAC-T	Incubation with AFB1 significantly decreased cell proliferation in a dose-dependent manner. Since the ratio of cells in sub-G1, S and G2/M phases was elevated, it was assumed that AFB1 inhibited cell proliferation by inhibiting the cell cycle. Flow cytometry also showed that incubation with AFB1 induced apoptosis in MAC-T cells.	[41]
high content screening	BF-2	AFB1 reduced cell viability in a dose-dependent manner, with IC50 estimated at 11.11 µM. Moreover, AFB1 generated strong oxidative stress.	[42]
Cell Proliferation Reagent WST-1	HepG-2, BEAS-2B	AFB1 decreased HepG-2 cell viability, with a IC50 estimated at 1 µM; however, after exposure of BEAS-2B cells to AFB1, cell viability was at 90% compared to the control group in all tested concentrations.	[43]
MTT	SK, MDCK, HeLa	Cytotoxicity was defined as a decrease in cell viability by 20%. This effect was achieved by incubating AFB1 for 24 h at a concentration of 25 µg/mL with HeLa cells, but it could not be achieved with other cells.	[31]
Triple Assay (AlamarBlue™, CFDA-AM and NRU)	RTGiLL-W1	Results showed that after 24 h of incubation, AFB1 and AFB2 were significantly cytotoxic towards mitochondria (with EC50 values of 36.34 and 97.1 µM, respectively) and significantly less cytotoxic towards plasma membrane (EC50 > 320.20 and 318.17 µM, respectively) and lysosomes (EC50 269.33 µM and >318.17 µM, respectively).	[44]
AlamarBlue™ assay	HepG-2, RAW 264.7	After 48 h of incubation with AFB1, the AlamarBlue™ assay showed a dose-dependent reduction in cell viability, with a IC50 of 3.12 ppm.	[45]
high content assay	HepG-2	Results showed that AFB1 deceased cell viability in a dose-dependent manner, with a significant increase in cell viability reduction at low concentrations and a similar reduction at the three highest concentrations tested.	[46]
MTT, LDH release	primary hepatocytes of *Cyprinus carpio*	The results of the MTT assay showed a time- and dose-dependent decrease in cell viability. Moreover, the activity of LDH in cultured medium was elevated after incubation with AFB1 compared to the control group.	[47]

**Table 4:** Cytotoxicity of ochratoxin A tested on different cell lines.

Type of Test	Cell Line	Results	Reference
MTT	Caco-2	Results after 24, 48 and 72 h of incubation showed that OTA inhibited cell viability in a dose- and time-dependent manner, which was comparable to the cytotoxicity of AFM1.	[34]
MTT	HK-2, HepG-2	OTA caused a significant viability decrease in HK-2 cells and HepG-2 cells in a dose-dependent manner in all tested concentrations.	[48]
CCK-8, flow cytometry, western blot	Het-1A	OTA at concentrations of 5 µM and 10 µM caused reductions in Het-1A cells after 24 h of incubation. The results also showed an increase in apoptosis cell ratio via flow cytometry. Western blot indicated increased expression levels of Bax, cleaved caspase-3, cleaved caspase-9 and cytochrome c, as well as decreased expression levels of Bcl-2, Bcl-xl, caspase-3 and caspase-9, which is typical for apoptosis.	[49]
MTT, NRU	Caco-2, Raw264.7, MDBK	After 48 h of incubation with OTA at 10 µg/mL concentration, cell viability was decreased by 7, 33 and 55% in Caco-2, Raw264 and MDBK cells, respectively, which was confirmed by both assays.	[38]
CCK-8, flow cytometry	BRL	After incubation with OTA at concentrations between 17 and 30 µM, cells’ viability decreased in a dose-dependent manner, with IC50 = 37.8 µM. Additionally, flow cytometry showed an increased ratio of apoptotic cells after incubation with OTA.	[50]
Cell Proliferation ELISA BrdU Kit	MAC-T	OTA inhibited cell proliferation in a dose-dependent manner. In a further analysis, OTA inhibited the cell cycle via the degradation of CDK4 and cyclin D1. In addition, OTA at a concentration of 1 µM caused an increase in apoptotic cell ratio by approximately 1.76-fold when compared to the control group.	[51]
MTT	HepG-2	Decrease in cell viability in a time- and dose-dependent manner, with IC50 values of 8.89, 3.58 and 1.86 µM for incubation periods of 24, 48 and 72 h, respectively.	[52]
MTT	Caco-2	Dose-dependent decrease in cell viability after 24 h of incubation with OTA at concentration ≥ 40 µM. The IC50 for OTA was calculated at 145.36 µM.	[28]
MTT	PBM	OTA at concentration of 12.5 mg/L significantly decreased both cell metabolic activity and proliferation (37.6 and 42.8%, respectively, compared to the control).	[53]
AlamarBlue™, CFDA-AM, NRU	RTH-149, PLHC-1, H4IIE	OTA had no strong cytotoxic properties against the tested cell lines, with IC50 values for NRU of 5.47 and 21.65 µg/mL for PLHC-1 and RTH-149, respectively (the remaining assays showed IC50 > 40.4 µg/mL for all cell lines).	[54]
MTT	K562, PBL, PBG	Significant decrease in K562 cell viability between 16 and 48 h of exposure at a concentration of 25 µg/mL. This decrease in cell viability was dose-dependent, which could also be observed in PBL and PBG cells incubated with OTA (although the cytotoxicity of OTA against these cell lines was lower).	[55]
Triple Assay (AlamarBlue™, CFDA-AM, NRU)	RTGiLL-W1	After 24 h of incubation, OTA had a similar cytotoxicity towards mitochondria (EC50 106.12 µM) and lysosomes (EC50 108.84 µM) and a significantly lower cytotoxicity towards plasma membrane (EC50 > 247.65 µM).	[44]
MTT	HepG-2	Significant, dose-dependent decrease in cell viability, GSH and MDA levels and GSH-Px activity.	[56]
MTT, NRU	RPTC, HKC, PKC, LLC-PK1	NRU assay showed that after 24 and 48 h of incubation, the viability of RPTC and HKC cells decreased, with a significant difference between males and females (from which cells were isolated); however, after 72 and 96 h, this difference was no longer visible. In PKC cells, OTA exhibited a similar cytotoxicity across all incubation periods in males and a slightly higher cytotoxicity in females after 48 h of incubation. Moreover, in LLC-PK1 cells, OTA caused a similar cytotoxicity across all incubation times. The MTT assay supported these results; however, it was less sensitive than the NRU assay.	[57]
MTT, flow cytometry	HEK 293	Dose-dependent decrease in cell viability, with a IC50 of 16 µM. In addition, flow cytometry showed that OTA caused a significant accumulation of cells in the S phase, thus disrupting the cell cycle.	[58]
MTT	PK15	OTA significantly increased the cell death ratio after 48 h of incubation. This study also showed a decrease in the concentration of thiol groups (SH) as well as a down-regulation of Hsp70 and Hsp27 expression.	[59]
MTT, AO/EB staining	HepG-2	After 24 h of incubation, OTA in a concentration range of 0–300 µM significantly decreased cell viability in a dose-dependent manner, with a IC50 of 210 µM. Additionally AO/EB staining revealed an increased ratio of apoptotic and necrotic cells (with a significant majority of apoptotic cells).	[60]
MTT	Vero	Significant decrease in cell viability, even at low concentrations, with a IC50 of 37 µM.	[61]
CCK-8, CASY cell counter assay	IHKE	OTA exhibited cytotoxic effects on IHKE cells, with an EC50 of 69.8 and 450.5 nmol/L (for CCK-8 and CASY assays, respectively).	[62]

**Table 5:** Cytotoxicity of fumonisins tested on different cell lines.

Type of Test	Cell Line	Results	Reference
MTT, LDH release	BRL 3A	Exposure to fumonisin FB1 resulted in slightly increased cell viability, even at high concentrations. The LDH activity was also unaffected by fumonisin FB1.	[35]
MTT	HK-2, HepG-2	No significant decrease in cell viability in HK-2 and HepG-2 cells in the presence of fumonisin B1.	[48]
MTT	CHO-K1, V79, C5-O, Caco-2, HepG-2	FB1 exhibited cytotoxic properties against all studied cell lines (sensitivity to FB1 after 72 h of exposure to 100 µg/mL: HepG-2 > V79 > CHO-K1 > C5-O > Caco-2). The CHO-K1 exhibited the highest sensitivity to FB1 at high concentrations, whereas V79 cells showed the highest sensitivity to FB1 at low concentrations (<25 µg/mL).	[63]
MTT, NRU	Caco-2, Raw264.7, MDBK	FB1 exhibited cytotoxic properties against raw264.7 cells, reducing cell viability by 25% after 48 h of incubation with FB1 at a concentration of 10 µg/mL. No significant decrease in cell viability was observed in MDBK and caco-2 cell lines.	[36]
MTT, LDH release	PK-15	FB1 exhibited weaker cytotoxic properties than the other tested mycotoxins (ZEN, AFB1, DON). After 48 h of incubation with FB1 (250 µM), cell viability was over 65%. Regarding the LDH release, the PK-15 cell line exhibited a significant decrease (contrary to the other tested mycotoxins) in LDH release after 24 h of incubation with FB1.	[40]
CCK-8, LDH release, flow cytometry	GES-1	FB1 decreased cell viability in a dose- and time-dependent manner. Highest results were observed after 48 h of incubation with FB1 at a concentration of 40 µM. Moreover, flow cytometry showed a significant increase in apoptotic cell number compared to the control group.	[64]
MTT	IPEC-J2	Results showed that IPEC-J2 cells are generally resistant to FB1 at concentrations from 5 to 40 µM, with no statistically significant decrease in cell viability after 48 h of incubation.	[65]
Cell Proliferation Reagent WST-1	HepG-2, BEAS-2B	At low concentrations, FB1 increased HepG-2 cell viability, whereas concentrations >100 µM resulted in a decrease in cell viability, with a IC50 of 399.2 µM. The FB1 exhibited cytotoxicity against BEAS-2B cells at concentrations >100 µM, with a IC50 of 355.1 µM.	[43]
MTT	PBM	FB1 at a concentration of 125 mg/L significantly decreased both cell metabolic activity and proliferation (55.6 and 56.1%, respectively, compared to the control).	[53]
MTT	SK	Cytotoxicity was defined as a decrease in cell viability by 20%. This effect could, however, not be achieved within the tested concentration range after 24 h of incubation for either FB1 or FB2.	[31]
Triple Assay (AlamarBlue™, CFDA-AM and NRU)	RTGiLL-W1	After 24 h of incubation, the results showed that FB1 and FB2 are not cytotoxic against RTGiLL-W1 cells.	[44]
CCK-8	porcine lymphocytes	After 24, 48 and 72 h of incubation, FB1 reduced cell viability in a time- and dose-dependent manner, but the IC50 could only be calculated for an incubation period of 72 h (IC50 = 101.15 µg/mL). This suggests that the cytotoxicity of FB1 against porcine lymphocytes is generally weak.	[66]

**Table 6:** Cytotoxicity of deoxynivalenol tested on different cell lines.

Type of Test	Cell Line	Results	Reference
MTT	HepG-2	Cell viability decreased from 20 to 53%, from 50 to 84% and from 48 to 72% at incubation periods of 24, 48 and 72 h, respectively.	[21]
MTT, LDH release	BRL 3A	Cell viability decreased in a dose- and time-dependent manner. Results also showed that DON increased LDH activity, apoptotic cell ratio and ROS production.	[35]
MTT, NRU	Caco-2	DON exhibited cytotoxic properties cells at a concentration of 1 µM. This effect was dose-dependent. Results also showed DON inhibited protein and DNA synthesis in a concentration-dependent manner, with IC50 values of 5 and 1.7 µM for protein and DNA synthesis, respectively. The production of MDA (lipid peroxidation marker) was also increased in the presence of DON, indicating increased oxidative stress in cells.	[67]
NRU	IPEC-12	Significant drop in proliferative cell viability at a concentration of 0.1 mg/mL after 24 h; however, no significant viability decrease was observed in differentiated cells, even after 24 h of incubation with DON at a concentration of 10 mg/mL. An increase in the ratio of apoptotic cells was also confirmed.	[68]
MTT	Jurkat T	The viability of Jurkat T cells decreased in a dose-dependent manner, with a IC50 of 4.7 (±6.15) µM after 48 h of incubation.	[69]
MTT	CHO-K1, V79, C5-O, Caco-2, HepG-2	DON exhibited cytotoxic properties against all studied cell lines (sensitivity to DON: CHO-K1 >V79 > C5-O > Caco-2 > HepG-2), with IC50 values ranging from 0.27 to 8.6 µg/mL after 48 h of incubation.	[63]
MTT, LDH release	PK-15	DON exhibited dose- and time-dependent cytotoxic properties. The MTT test showed that after 48 h of incubation, the IC50 for DON was estimated at 3.6 µM. Regarding the LDH release, DON at a concentration of 2.4 µM caused an increase in LDH release by 30% after 24 h of incubation.	[40]
MTT	IPEC-J2	DON reduced IPEC-J2 cell viability in a dose-dependent manner after 48 h of incubation. Significant results were observed at a concentration of 1 µM, with a maximum decrease in cell viability at 2 µM (viability 38% compared to the control). The IC50 for DON was calculated at 1.83 µM.	[65]
high content screening	BF-2	DON reduced cell viability in a dose-dependent manner, with a IC50 estimated at 15.96 µM. Moreover, DON induced the highest levels of intracellular calcium (Ca^2+^).	[42]
MTT, NRU	Vero	Significant decrease in cell viability in a dose- and time-dependent manner. The IC50 calculated from the MTT assay ranged from 5.05 to 8.02 µM, whereas IC50 calculated based on the NRU assay ranged from 3.33 to 10.0 µM.	[70]
AlamarBlue™, CFDA-AM, NRU	RTH-149, PLHC-1, H4IIE	DON had strong cytotoxic properties against PLHC-1 and RTH-149 cell lines, with IC50 values of 2.65, 10.51 and 1.52 µg/mL for PLCH-1 cells (for AlamarBlue™, CFDA-AM and NRU, respectively) and >29.6, 16.31 and 1.2 µg/mL for H4IIE cells (for AlamarBlue™, CFDA-AM and NRU, respectively). The IC50 for all three tests for RTH-149 was >29.6 µg/mL, suggesting a low cytotoxicity against this cell line.	[54]
MTT	K562, PBL, PBG	Significant decrease in K562 cell viability for 4–48 h of exposure at a concentration of 25 µg/mL. Moreover, DON was cytotoxic against PBL and PBG cells, although not as strongly as against K562. In the case of all three cell lines, the decrease in cell viability was dose-dependent.	[55]
MTT	SK, MDCK, HeLa	Cytotoxicity was defined as a decrease in cell viability by 20%. This effect was achieved by incubating DON for 24 h at a concentration 100 µg/mL with HeLa cells and at a concentration of 0.8 µg/mL with SK cells, but it could not be achieved with MDCK cells.	[31]
Triple Assay (AlamarBlue™, CFDA-AM, NRU)	RTGiLL-W1	DON was cytotoxic against lysosomes (EC50 174.07 µM), with a significantly lower cytotoxicity against plasma membrane (EC50 > 337.47 µM) and mitochondria (EC50 > 337.47 µM).	[44]
NRU, SRB, WST-1	RTgill-W1, IPEC-1, IPEC-2, HepG-2	NR and SRB assays showed that DON reduced RTgill-W1 cell viability. At a concentration 40 µmol/L, viability was reduced by 63 and 52% (SRB and NR assay, respectively). Regarding IPEC-1 and IPEC-2 cells, DON decreased cell viability in a dose-dependent manner in both lines. The WST-1 assay showed that at a concentration 6.9 µmol/L, viability was reduced by 65.6 and 60.9% (for IPEC-1 and IPEC-2 cells, respectively). In HepG2, DON caused a dose-dependent decrease in cell viability. The WST-1 assay showed that at a concentration 3.5 µmol/l, viability was decreased by 39.7%.	[71]
MTT	SK, VERO, MDCK, BEL	Dose- and time-dependent effect on the viability of MDCK cells was observed. For higher concentrations, after 16 and 24 h, a significant decrease in cell viability was observed, whereas at lower concentrations, after 2 and 24 h of incubation, the presence of DON resulted in an increased cell viability. In SK cells, DON at a concentration of 100 µg/mL resulted in a constant decrease in cell viability (up to 25% of control), whereas concentrations of 0.1–10 µg/mL had less pronounced effects after 2 and 16 h and stimulated cell viability after 24 h of incubation (up to 125% of the control). In VERO cells, all concentrations caused a slight decrease in cell viability, followed by recovery after 4 h and decrease after 16 and 24 h at concentrations between 1 and 100 µg/mL. In BEL cells, ZEN at all concentrations resulted in a significant reduction in cell viability after 2 and 4 h, followed by recovery at low concentrations and continued decrease at higher concentrations.	[72]
AlamarBlue™	HepG-2, RAW 264.7	After 48 h of incubation, AlamarBlue™ assay showed a dose-dependent reduction in cell viability, with a IC50 of 0.23 ppm.	[45]
high content assay	HepG-2	After 24 h of incubation, DON decreased cell viability in a dose-dependent manner, with a significant increase in cell viability reduction at low concentrations and a similar reduction at the three highest concentrations tested.	[46]
MTT, NRU	Caco-2	At concentrations between 7.5 and 6.67 µM after 48 h of incubation, a dose-dependent decrease in cell viability in both assays was observed, with a IC50 calculated at 1.39 µM for MTT and at 1.19 µM for the NRU assay.	[73]
CCK-8	porcine lymphocytes	After 24, 48 and 72 h of incubation, DON reduced cell viability in a time- and dose-dependent manner, with IC50 values of 0.43, 0.41 and 0.31 µg/mL after 24, 48 and 72 h of incubation, respectively.	[66]
MTT, LDH release	primary hepatocytes of *Cyprinus carpio*	Time- and dose-dependent decrease in cell viability was observed. Moreover, the activity of LDH in the culture medium was elevated after incubation with DON compared to the control group.	[47]
MTT	IPEC-1	After 24 h of incubation with DON, the cells exhibited a dose-dependent decrease in viability, with IC10 and IC80 values of 0.31 and 16.54 µM, respectively.	[74]
AlamarBlue™, BrdU	HepG-2, MRC-5	The results of the AlamarBlue™ assay showed that MRC-5 cells were more susceptible to DON, with IC50 values of 0.65 and 1.4 µM for MRC-5 and HepG-2 cells, respectively. However, the results of the BrdU test showed that HepG-2 cells were more susceptible, with IC50 values of 5.3 and 3.5 µM for MRC-5 and HepG-2 cells, respectively.	[75]

**Table 7:** Cytotoxicity of T-2 and HT-2 toxins tested on different cell lines.

Type of Test	Cell Line	Results	Reference
CCK-8	porcine Leydig cells	After 24 h of incubation; the IC50 values were 0.0209 and 0.0401 µM for T-2 and HT-2 toxin, respectively. Cytotoxic effect was dose-dependent.	[76]
MTT	HepG-2	The IC50 values obtained by the MTT assay were 68.6 ± 4.8 nM and 61.9 ± 2.4 nM at 24 and 48 h, respectively; T-2 toxin decreased cell viability in a dose- and time-dependent manner.	[77]
MTT, LDH release	SerW3	That T-2 toxin exhibits cytotoxic properties in a dose- and time-dependent manner. Cytotoxicity was assessed after 24 and 48 h of incubation at concentrations of 12, 120 and 1200 ng/mL. The LDH cytotoxicity assay also confirmed cell membrane damage in cells exposed to T-2 toxin, suggesting that T-2 toxin has cytotoxic properties.	[78]
MTT, LDH release	TM3 Leydig cells	T-2 decreased cell viability in a dose-dependent manner. Cell viability was decreased to 82.95, 63.47 and 32.79% for 1, 10 and 100 nM, respectively, compared to the control. Moreover, T-2 toxin increased the LDH release at concentrations of 10 and 100 nM. Flow cytometry also showed an increased ratio in apoptotic cells after incubation with T-2 toxin.	[79]
MTT, NRU	Vero	After 24 h of incubation at concentrations of 0–100 nM and 0–12 nM (for MTT and NRU, respectively), the assays showed growth inhibition from 0 to 70.7 ± 2.9% and from 0 to 85.3 ± 4.2% in a dose-dependent manner for MTT and NRU assays, respectively.	[80]
MTT	HepG-2	T-2 toxin decreased cell viability (by 2 to 66%) in a dose-dependent manner, without a significant difference among differed exposure times (24, 48 and 72 h). The IC50 for T-2 toxin was measured at 38 nM after 24 h of incubation.	[21]
Caspase-3 activity, Hoechst 33,258 dye staining	RPTCE, NHLF	Both T-2 and HT-2 toxin exhibited a similar cytotoxicity, with IC50 values of 0.2 and 0.8 µM for T-2 and HT-2 toxin, respectively, in the RPTEC cell line and 0.5 and 0.7 µM for T-2 and HT-2 toxin, respectively, in the NHLF cell line. Based on increased caspase-3 activity and the results from staining with Hoechst 33,258 dye, T-2 and HT-2 toxins promoted apoptotic cell death in both cell lines.	[81]
CCK-8	TM4	Results showed dose-dependent decrease in cell viability, with a IC50 of 8.1 nM after 24 h of incubation. Further analyses of apoptosis rate, caspase-3, caspase-8 and caspase-9 activity showed that T-2 toxin promoted apoptotic cell death. Moreover, ROS levels were significantly increased, and antioxidant enzymes levels were decreased in cells incubated with T-2 toxin.	[82]
MTT, NRU	Vero	After 24, 48 and 72 h of incubation, a significant dose- and time-dependent decrease in cell viability was observed. The IC50 calculated based on the MTT assay ranged from 7 to 12 nM, whereas that calculated based on the NRU assay ranged from 4 to 5 nM.	[70]
MTT	SK, MDCK, HeLa	Cytotoxicity was defined as a decrease in cell viability by 20%. This effect was achieved by incubating T-2 toxin for 24 h at a concentration 100 µg/mL with HeLa cells and at a concentration 0.4 µg/mL with SK cells, but it could not be obtained with MDCK cells. Additionally, the cytotoxicity of HT-2 toxin was tested against SK cells; 20% decrease in cell viability after 24 h of incubation with HT-2 toxin was observed at a concentration of 3.1 µg/ml.	[31]
AlamarBlue™ BrdU	HepG-2, MRC-5	The results of the AlamarBlue™ assay showed that MRC-5 cells were more susceptible to T-2 toxin, with IC50 values estimated at 0.00341 and 0.008 µM for MRC-5 and HepG-2 cells, respectively. The results of the BrdU test supported this, with IC50 values calculated at 0.0035 and 0.0085 µM for MRC-5 and HepG-2 cells, respectively.	[75]

**Table 8:** Cytotoxicity of zearalenone tested on different cell lines.

Type of Test	Cell Line	Results	Reference
MTT	Caco-2	After 24, 48 h and 72 h of incubation, ZEN exhibited cytotoxic properties in a concentration- and time-dependent manner, although not as strongly as OTA or AFM1.	[34]
MTT, LDH release	BRL 3A	ZEN exhibited cytotoxic properties in a dose- and time-dependent manner, but not as strongly as DON or AFB1. After 24 h, the activity was increased in a dose-dependent manner. However, ROS production and apoptotic cell ratio were not affected.	[35]
NRU, MTT	Caco-2	The IC50 for ZEN was 15 µM in the NRU assay and 25 µM in the MTT test. In addition, ZEN inhibited protein and DNA synthesis. The IC50 values were 19 and 10 µM for protein and DNA synthesis, respectively.	[67]
MTT	Cheng liver cell	ZEN inhibited Cheng liver cell proliferation in a dose-dependent manner. Cytotoxicity was assessed after 6, 12 and 24 h. After 12 and 24 h, cell viability was reduced by 19.3 and 41.5%, respectively (concentration 0–200 µM). The IC50 was ~100 µM after 12 and 24 h.	[83]
MTT	CHO-K1	Cytotoxicity was assessed after 24, 48 and 72 h at concentrations of 12.5, 18.75, 25, 37.5, 50, 75 and 100 µM. The IC50 was >100, 60.3 and 55 µM after 24, 48 and 72 h of incubation, respectively.	[84]
MTT, NRU	CHO-K1	ZEN decreased cell viability in a dose-dependent manner, with IC50 values of 108.76 and 79.4 µM, respectively, after 24 h of incubation. The ROS concentration was also significantly increased after incubation with ZEN.	[23]
MTT	CHO-K1, V79, C5-O, Caco-2, HepG-2	ZEN exhibited cytotoxic properties against all studied cell lines (sensitivity to ZEN: C5-O > Caco-2 > HepG-2 > V79 > CHO-K1). After 48 h of incubation with ZEN at 100 µg/mL, cell proliferation was inhibited by 46, 41, 33, 27 and 24%, respectively.	[63]
MTT, LDH release	PK-15	ZEN exhibited dose- and time-dependent cytotoxic properties. After 48 h of incubation, the IC50 for ZEN was estimated at 121.8 µM. Regarding the LDH release, at ZEN concentration of 84.2 µM caused an increase in LDH release to 130% after 24 h of incubation.	[40]
MTT	HepG-2	Decrease in cell viability in a time- and dose-dependent manner was observed. The IC50 values were 55.79, 39.88 and 29.48 µM for incubation times of 24, 48 h and 72 h, respectively.	[52]
MTT	IPEC-J2	In contrast to other similar studies, ZEN significantly increased IPEC-J2 cell viability at a concentration of 10 µM after 48 h of incubation and significantly decreased cell viability at 40 µM.	[65]
high content screening	BF-2	ZEN reduced cell viability in a dose-dependent manner, with an estimated IC50 of 170.24 µM.	[42]
NRU	HepG-2	Decrease in cell viability after 72 h of incubation, with an estimated IC50 of 54.6 µM observed.	[85]
MTT	K562, PBL, PBG	Significant decrease in K562 cell viability between 16 and 48 h of exposure at a concentration of 25 µg/mL was observed. Incubation with concentrations of 12.5–25 µg/mL resulted in a decrease in cell viability in K562 and PBL cells but not in PBG cells, suggesting that ZEN is less cytotoxic that the other tested mycotoxins (DON and OTA).	[55]
MTT	SK, MDCK, HeLa	Cytotoxicity was defined as a decrease in cell viability by 20%. This effect could not be achieved in any of the three cell lines after 24 h of incubation at concentrations within the tested range (0.01–100 µg/mL cell culture medium).	[31]
Triple Assay (AlamarBlue™, CFDA-AM, NRU)	RTGill-W1	ZEN was primarily cytotoxic against lysosomes (EC50 25.19 µM), followed by plasma membrane (EC50 71.48 µM) and mitochondria (EC50 135.14 µM).	[44]
MTT	SK, Vero, MDCK, BEL	At low concentrations, ZEN had little or no effect on cell viability after 2 and 24 h of incubation. At the higher concentration (100 µg/mL), a significant drop in cell viability was observed after 16 h (up to 40% of the control). In MDCK cells, 100 µg/mL of ZEN resulted in a significant decrease in cell viability after 2 and 16 h of incubation, but after 24 h, viability reached a level of 110% compared to the control. At lower concentrations, ZEN slightly stimulated cell viability after 2 and 24 h of incubation. In SK cells, a high ZEN concentration resulted in a significant drop in cell viability (up to 20% of the control), whereas at lower concentrations, ZEN stimulated cell viability (up to 160% of the control after 16 h).	[72]
MTT	HepG-2	Significant, dose-dependent decrease in cell viability, GSH content and GSH-Px activity was observed.	[56]
AlamarBlue™	HepG-2, RAW 264.7	After 48 h of incubation, the AlamarBlue™ assay showed a dose-dependent reduction in cell viability, with a IC50 calculated at 6.45 ppm.	[45]
high content assay	HepG-2	ZEN decreased cell viability in a dose-dependent manner, with a significant increase in cell viability reduction between concentrations of 5.77 × 10^−2^ μg/mL and 6.41 × 10^−3^ μg/mL and a similar reduction at the two highest concentrations tested.	[46]
CCK-8	porcine lymphocytes	ZEN reduced cell viability in a time- and dose-dependent manner, with calculated IC50 values of 19.55, 20.6 and 16.6 µg/mL after 24, 48 and 72 h of incubation, respectively.	[66]
NRU, MTT	RTL-W1, RTGill-W1, SHK-1, RT-EQ clone 8, CCB	ZEN caused a biphasic response in SHK-1, CCB8 and RTL-W1 cells (increase in cell viability at lower concentrations and decrease at higher concentrations), whereas in RT EQ clone 8 and RTgill-W1, it caused a decrease in cell viability at concentrations ≥1250 ng/mL and ≥625 ng/mL, respectively. On the other hand, the MTT assay showed a biphasic response in all cell lines.	[86]

**Table 9:** Cytotoxicity of citrinin tested on different cell lines.

Type of Test	Cell Line	Results	Reference
MTT	SK	Cytotoxicity was defined as a decrease in cell viability by 20%. However, this effect could not be achieved within the tested concentration range after 24 h of incubation.	[31]
MTT	PBM	CIT at a concentration of 125 mg/L significantly decreased both cell metabolic activity and proliferation (41.8 and 44.2%, respectively, compared to the control).	[53]
MTT, flow cytometry	HEK 293	Dose-dependent decrease in cell viability, with a IC50 estimated at 189 µM observed. In addition, flow cytometry showed that CIT caused a significant accumulation of cells in the G2/M phase, thus disrupting the cell cycle.	[58]
MTT	PK-15	CIT significantly increased the cell death ratio after 48 h of incubation. This study also showed a decrease in the concentration of thiol groups (SH) and an up-regulation of Hsp70 and Hsp27 expression.	[59]
Nuclei counted via hemocotometer	MDBK, PFBK	The viability of MDBK cells was significantly reduced after 24 h of incubation with CIT at concentrations of 150–300 µM (IC50 calculated at 140 µM). In PFBK cells, CIT caused a significant decrease in cell viability after 24 h when used at concentrations of 0.5–1 mM (IC50 calculated at 380 µM).	[87]
MTT, AO/EB staining	HepG-2	CIT in a concentration of 0–300 µM significantly decreased cell viability in a dose-dependent manner, with a IC50 calculated at 155 µM. Additionally, AO/EB staining revealed an increased ratio of apoptotic and necrotic cells.	[60]
MTT	Vero	Significant decrease in cell viability, albeit at relatively high concentrations (no effects were visible at concentrations ≤ 60 µM), was observed. The IC50 was estimated at 220 µM.	[61]
MTT and trypan blue exclusion assay	HL-60	After 24 h of incubation, a significant decrease in cell viability was observed at concentrations ≥ 50 µM (no significant effect was observed at a concentration of 25 µM). Cell viability was reduced to 11.9% compared to the control at 100 µM (MTT assay).	[88]
MTT	human osteoblasts	Decrease in cell viability by 40–70% compared to the control was observed. Moreover, the JNK pathway (which is essential for apoptosis in some cell types) was activated in a dose-dependent manner after incubation with CIT for 1 h.	[89]
CCK-8, CASY cell counter assay	IHKE	CIT exhibited cytotoxic effects, with EC50 values calculated at 56.3 and 27.7 µmol/L (for CCK-8 and CASY assays, respectively). Moreover, a significant increase in caspase-3 activity (indicator of apoptosis) was observed after incubation with CIT at concentrations ≥ 5 µmol/L.	[62]
AlamarBlue™	A549	CIT reduced A549 cell viability in a dose- and time-dependent manner. After 6 h of incubation with 50 µg/mL, CIT viability dropped to 80% of that of the control, whereas after 24 h and 72 h, it dropped to 40 and 20% of that of the control, respectively.	[90]

**Table 10:** Cytotoxicity of enniatins tested on different cell lines.

Type of Test	Cell Line	Results	Reference
MTT	Caco-2	After 24 h of incubation, the IC50 value for ENA1 was 14.8 µM, whereas those for ENA, ENB and ENB1 were not within the tested concentration levels. However, after 48 and 72 h, all enniatins (except ENB) exhibited cytotoxic properties, with IC50 values after 48 h of 6.8, 7.7, 11.3 for ENA, ENA1, ENB1, respectively, and after 72 h of 1.6, 1.3, 2.8, 11.7 for ENA, ENA1, ENB1 and ENB, respectively.	[91]
Triple Assays (AlamarBlue™, CFDA-AM, NRU)	RTGill-W1	ENs were primarily cytotoxic against lysosomes (EC50 4.24, 5.7, 26.62 and 8.47 µM for ENA, ENA1, ENB and ENB1, respectively), followed by mitochondria (EC50 12.37, 11.34, 112.19 and 34.5 µM for ENA, ENA1, ENB and ENB1, respectively) and plasma membrane (EC50 11.07, 16.75, >156.29 and 77.00 µM for ENA, ENA1, ENB and ENB1, respectively).	[44]
Flow cytometry	IPEC-J2	Flow cytometry showed that ENA1 was the most cytotoxic enniatin, whereas ENAB was the least cytotoxic one. The cytotoxicity of enniatins followed the order ENA > ENA1 > ENB1 > ENB (30, 86, 93, 95% of viable cells compared to the control, respectively).	[92]
AlamarBlue, BrdU	HepG-2, MRC-5	AlamarBlue™ assay showed that MRC-5 cells were more susceptible to enniatins, with IC50 for MRC-5 estimated at 3.65, 6.4, 5.85 and 4.6 µM (for ENA, ENA1, ENB and ENB1, respectively) and for HepG-2 at 8.35, 14.9, 321.3 and 22.6 µM (for ENA, ENA1, ENB and ENB1, respectively). This is supported by the results from the BrdU test, albeit with the exception of enniatin B. The IC50 values for MRC-5 were estimated at 0.7, 1.25, 2.25 and 1.3 µM (for ENA, ENA1, ENB and ENB1, respectively) and for HepG-2 at 2.05, 3.4, 1 and 3.15 µM (for ENA, ENA1, ENB and ENB1, respectively). Additionally, the cytotoxicity of ENB2 and ENB3 against HepG-2 cells was tested via the BrdU assay, resulting in IC50 values of 8.45 and 8.9 µM for ENB2 and ENB3, respectively.	[75]
MTT	Caco-2	ENA and ENA had little to no effect on Caco-2 cell viability, whereas ENA1 and ENB1 decreased cell viability in a dose-dependent manner, with IC50 values of 12.3 and 19.5 µM, respectively.	[93]
MTT	Caco-2, HepG-2, HT29	Almost all examined enniatins decreased cell viability in a dose-dependent manner. After 48 h of incubation, the IC50 values for Caco-2 cells were 9.3, 2.7, 2.6, 5.3 and 2.9 µM (for ENA, ENA1, ENA2, ENB1 and ENB4, respectively), whereas those for HT29 were 8.2, 1.4, 2.8, 3.7 and 15 µM (for ENA, ENA1, ENB, ENB1 and ENB4, respectively). The IC50 values for HepG-2 cells were 4.6, 2.6, 8.5 and 12.7 µM (for ENA, ENA1, ENB1 and ENB4, respectively), and those for ENJ3 could not be calculated in any of the cell lines; also, the IC50 values for ENA2 in HT29 cells, ENB in Caco-2 cells and ENA2, ENB in HepG-2 cells could not be determined.	[94]
MTT	Jurkat-T	ENB at a concentration of 15 µM decreased cell viability by 21, 23 and 29% compared to the control after 24, 48 and 72 h of incubation, respectively. Additionally, after 48 h of incubation, the ratio of apoptotic and necrotic cells was significantly increased compared to the control.	[95]
MTT	CHO-K1	ENA, ENA1, ENB and ENB1 decreased cells’ viability in a dose- and time-dependent manner. After 24 h of incubation, the IC50 values were >7.5, 8.8, 11 and 4.53 µM for ENA, ENA1, ENB and ENB1, respectively. These values decreased after 72 h to 3.33, 1.65, 2.8 and 2.47 µM, respectively.	[96]
AlamarBlue™, NRU	RAW 267.4	ENB decreased cell viability in a dose-dependent manner. The IC50 values were 2.6 and 4.7 µM for AlamarBlue™ and NRU, respectively. Additionally, ENB at a concentration of 1.25 µM or higher caused an increase in the ratio of cells in G1 phase, thus suggesting cell cycle inhibition. Moreover, ENB also increased the ratio of apoptotic and necrotic cells.	[97]

**Table 11:** Cytotoxicity of combinations of two mycotoxins tested on different cell lines.

Type of Test	Cell Line	Combinations	Results	Reference
MTT	HepG-2	DON + PAT PAT + T2 DON + T-2	The combined cytotoxicity of DON + PAT was similar to that of DON and PAT applied individually. The combination of PAT + T-2 reduced cell viability by 32 to 88% compared to T-2 toxin and by 45 to 95% compared to PAT. The combination of DON + T-2 reduced cell viability by up to 48% compared to T-2 and by 63% compared to DON after 24 h of incubation; however, after 72 h of incubation, the cytotoxicity of individual mycotoxins and their combination was roughly the same.	[21]
MTT	Caco-2	AFM1 + OTA AFM1 + ZEN	Combination of AFM1 + OTA exhibited stronger cytotoxic properties than each mycotoxin applied individually after 24 and 72 h; however, the cytotoxicity of a combination of AFM1 + ZEN was roughly the same as that of individual mycotoxins after 72 h of incubation.	[34]
MTT, LDH release	BRL 3A	DON + AFB1 ZEN + AFB1 DON + ZEN	Combinations of DON + AFB1 and ZEN + AFB1 exhibited stronger cytotoxic properties than the individual mycotoxins; in addition, DON + ZEN was more cytotoxic than ZEN + AFB1.	[35]
MTT	H-2, HepG-2	OTA + FB1	In HK-2 cells, co-exposure to FB1 slightly increased OTA cytotoxicity.	[48]
CCK-8	Leydig cells	T2 + HT-2	Combination of T-2 and HT-2 toxins exhibited stronger cytotoxic properties than T-2 and HT-2 toxins applied individually, except for the highest concentration.	[76]
MTT, NRU	MDBK	AFB1 + FB1, OTA + FB1, OTA + AFB1	The reduction in cell viability assessed by the MTT assay reached up to 25, 19 and 8% for OTA + FB1, AFB1 + FB1 and OTA + AFB1, respectively. The results obtained via the neutral red assay confirmed these trends, albeit not as strongly as the MTT.	[36]
MTT	HepG-2	OTA + ZEN	Decrease in cell viability in a time- and dose-dependent manner was observed. However, cytotoxicity was not as strong as that of OTA alone, indicating an antagonistic interaction of these two mycotoxins. The IC50 values were 34.25, 10.08 and 7.36 µM for incubation times of 24, 48 and 72 h, respectively.	[52]
MTT	Caco-2	PAT + OTA	OTA was predominantly responsible for the cytotoxicity of the mixture; however, at higher concentrations of patulin, the cytotoxicity of the mixture was lower than that of patulin and OTA alone, suggesting an antagonistic effect.	[28]
MTT	IPEC-J2	DON + FB1 DON + ZEN ZEN + FB1	In cytotoxic concentrations (measured previously for individual mycotoxins), there was no increased cytotoxicity in mixtures containing DON. A mixture of FB1 and ZEN was more cytotoxic than those two mycotoxins applied individually. At non-cytotoxic concentrations (measured for individual mycotoxins), all mixtures were cytotoxic against IPEC-J2 cells.	[65]
MTT	Caco-2	ENA + ENA1 ENA1 + ENB1 ENA1 + ENB ENA + ENB ENA + ENB1 ENB + ENB1	All binary mixtures reduced cell viability in a dose-dependent manner by 48, 47, 35, 33, 32 and 26% for ENA + ENA1, ENA1 + ENB1, ENA1 + ENB, ENA + ENB, ENA + ENB1 and ENB + ENB1, respectively.	[91]
high content screening	BF-2	ZEN + AFB1 DON + AFB1 DON + ZEN	ZEN + AFB1 and DON + AFB1 exhibit a higher cytotoxicity than their components tested individually, whereas the combination of DON + ZEN had a lower cytotoxicity than their components individually.	[42]
Cell Proliferation Reagent WST-1	HepG-2, BEAS-2B	FB1 + AFB1	At low concentrations, a combination of AFB1 + FB1 increased HepG-2 cell viability, whereas higher concentrations significantly decreased cell viability (up to 32% of the control group); there was a weak antagonistic interaction. This combination also exhibited cytotoxicity against BEAS-2B cells. Cell viability decreased significantly in all concentrations (up to 26% of the control group), with a strong additive interaction.	[43]
NRU	Vero	DON + T-2	This combination decreased cell viability in a dose-dependent manner and exhibited a strong antagonistic mode of interaction, even at low concentrations.	[70]
MTT	HepG-2	OTA + ZEN	The combined cytotoxicity of ZEN and OTA was higher than that of each mycotoxin tested individually. In addition, the oxidative damage caused by this combination was also greater.	[56]
AlamarBlue™	HepG-2, RAW 264.7	AFB1 + ZEN AFB1 + DON DON + ZEN	Combination of AFB1 + DON and ZEN + DON was more cytotoxic than each mycotoxin tested individually; however, in the case of AFB1 + ZEN, the IC50 values for component mycotoxins were higher than those of the individual mycotoxins, and change was observed in cell viability compared to individual mycotoxins.	[45]
high content assay	HepG-2	AFB1 + ZEN AFB1 + DON DON + ZEN	The combination of AFB1 + ZEN showed a similar cytotoxicity than its components (tested individually) at lower concentrations, but at the two highest concentrations, the cytotoxicity of the mixture was significantly higher. Similar results were observed for ZEN + DON and DON + AFB1 mixtures.	[46]
CCK-8	porcine lymphocytes	FB1 + ZEN FB1 + DON DON + ZEN	After 24 h of incubation, the mixtures did not exhibit any interactions, whereas after 48 h, the mixtures containing DON (DON + FB1 and DON + ZEN) started to exhibit antagonistic effects. After 72 h, this effect was even stronger, and additionally, FB1 + ZEN also started to exhibit an antagonistic effect.	[66]
MTT, LDH release	primary hepatocytes of *Cyprinus carpio*	DON + AFB1	Time- and dose-dependent decrease in cell viability, which was stronger than the decrease caused by the component mycotoxins (tested individually), was observed. Moreover, the activity of LDH in the culture medium after 4 h of incubation was significantly more elevated compared to the individual mycotoxins; however, after prolonged incubation, this difference was no longer visible.	[47]
MTT, flow cytometry	HEK 293	OTA + CIT	This mixture was more cytotoxic than its components tested individually (IC50 of 7 µM, compared to 16 and 189 µM for OTA and CIT, respectively). Additionally, flow cytometry showed that OTA caused a significant accumulation of cells in S and G2/M phases, thus disrupting the cell cycle.	[58]
MTT	PK-15	OTA + CIT	This combination significantly increased the cell death ratio after 48 h of incubation. This study also showed a decrease in the concentration of thiol groups (SH) after 12 h (compared to 24 h in the case of the individual mycotoxins), as well as a strong up-regulation of Hsp70 and Hsp27 expression.	[59]
MTT, AO/EB staining	HepG-2	CIT + OTA	The combination of OTA and CIT (42 and 31 µm, respectively (20% of respective IC50)) reduced cell viability by 50%, suggesting a synergic interaction. Moreover, AO/EB staining showed an increased ratio of apoptotic and necrotic cells (with the majority of apoptotic cells).	[60]
MTT	Vero	OTA + CIT	Increased cytotoxicity of this mixture compared to its components tested individually. The estimated IC50 value was 24 µM (compared to 37 and 220 µM for OTA and CIT, respectively), suggesting a synergic interaction between the components.	[61]
MTT	CHO-K1	ENA + ENA1 ENA + ENB ENA + ENB1 ENA1 + ENB ENA1 + ENB1 ENB + ENB1	After 24 h of incubation, the IC50 values were 0.78 M, 0.66 M, 0.94 M, 0.96 M, 0.44 M and 0.78 µM for ENA + ENA1, ENA + ENB, ENA + ENB1, ENA1 + ENB, ENA1 + ENB1 and ENB + ENB1, respectively. Mixtures of ENA + ENA1, ENA + ENB1, ENA1 + ENB and ENB + ENB1 exhibited additive cytotoxicity, whereas ENA + ENB and ENA1 + ENB1 exhibited synergic interactions.	[96]
Caspase-3 activity	IHKE	CIT + OTA	At lower concentrations, CIT had no effect on OTA-induced caspase-3 activity. At concentrations of 2.5 and 5 µmol/L, CIT exhibited antagonistic interactions, lowering the OTA-induced caspase-3 activity. At higher concentrations, however, this effect was additive.	[62]

**Table 12:** Cytotoxicity of combinations of three mycotoxins tested on different cell lines.

Type of Test	Cell Line	Combinations	Results	Reference
MTT	HepG-2	PAT + DON + T-2	The combination of PAT + DON + T-2 exhibited stronger cytotoxic properties than each toxin individually, irrespective of the concentration. This combination reduced cell viability by 30 to 57% compared to PAT, 12 to 46% compared to T-2 and 30 to 38% compared to DON applied individually.	[21]
MTT	Caco-2	AFM1 + OTA + ZEN	Combination of AFM1 + OTA + ZEN had stronger cytotoxic properties against Caco-2 cells than each mycotoxin applied individually.	[34]
MTT, NRU	Caco-2, RAW264.7, MDBK	OTA + AFB1 + FB1	After 48 h of incubation, concentration-dependent decrease in cells’ viability was observed. MDBK cells’ viability decreased by 26% and 14% (for MTT and NRU, respectively). There was no cytotoxic effect in raw264.7 cells, and viability of caco-2 cells decreased by 9% at highest tested concentration.	[36]
MTT	IPEC-J2	DON + FB1 + ZEN	At cytotoxic concentrations (measured for individual mycotoxins), the mixture was less cytotoxic than DON applied individually. However, at non-cytotoxic concentrations (measured for individual mycotoxins), the mixture was significantly more cytotoxic than the individual mycotoxins.	[65]
MTT	Caco-2		All tertiary mixtures reduced cell viability in a dose-dependent manner by approximately 40%	[91]
high content screening	BF-2	ZEN + DON + AFB1	This combination had a higher cytotoxicity than its components when tested individually.	[42]
MTT	PBM	OTA + FB1 + CIT	This combination exhibited stronger cytotoxic properties than any of these mycotoxins tested individually. It significantly decreased both cell metabolic activity and proliferation (31.2 and 33.1%, respectively, compared to the control).	[53]
AlamarBlue™	HepG-2, RAW 264.7	AFB1 + ZEN + DON	After 48 h of incubation, results showed that this combination exhibited stronger cytotoxic properties than its individual components against both cell lines.	[45]
high content assay	HepG-2	ZEN + DON + AFB1	After 24 h of incubation, this combination was more cytotoxic than its components tested individually at higher tested concentrations, whereas no differences in cytotoxicity were observed at lower concentrations (compared to individual mycotoxins).	[46]
CCK-8	porcine lymphocytes	DON + ZEN + FB1	This mixture exhibited a strong antagonistic effect across all incubation times (24, 58, 72 h).	[66]
MTT	CHO-K1	ENA +ENA1 + ENB, ENA + ENB + ENB1, ENA + ENA1 + ENB1, ENA1 + ENB + ENB1	After 24 h of incubation, the IC50 values were 0.61 M, 0.74 M, 0.66 M and 0.97 µM for ENA +ENA1 + ENB, ENA + ENB + ENB1, ENA + ENA1 + ENB1 and ENA1 + ENB + ENB1, respectively. A mixture of ENA1 + ENB + ENB1 exhibited additive cytotoxicity, whereas the remaining tertiary mixtures exhibited synergic interactions.	[96]

## Data Availability

Not applicable.

## Supplementary Materials

The following supporting information can be downloaded at: https://www.mdpi.com/article/10.3390/toxins14040244/s1, Table S1: Summary of reviewed literature, cell lines and cytotoxicity assays used and mycotoxins tested.
