# Supplementary material for: Cytotoxicity of Mycotoxins and Their Combinations on Different Cell Lines: A Review
Source: Toxins (Basel). 2022 Mar 30;14(4):244. doi: 10.3390/toxins14040244 (PMC9031280; doi:10.3390/toxins14040244)
Supplement: Supplementary file 1 [file toxins-14-00244-s001.zip › toxins-1611413-supplementary.pdf]

toxins

MDPI

Review

Cytotoxicity of Mycotoxins and Their Combinations on Different Cell Lines: A Review

Paweł Skrzydlewski, Magdalena Twarużek and Jan Grajewski

Table S1. Summary of reviewed literature, cell lines and cytotoxicity assays used, and mycotoxins tested.

MYCOTOXIN	CELL LINE	CYTOTOXICITY TEST	REFERENCE
SINGLE MYCOTOXIN			
Patulin			
	HepG-2	MTT	Fernández-Blanco et al. (2018)
	HepG-2	MTT	Ayed-Boussema et al. (2013)
	HepG-2	CCK-8	Zhang et al. (2022)
	HepG-2	MTT	Yang et al. (2018)
	CHO-K1	MTT, NR	Ferrer et al. (2009)
	CHO-K1	MTT	Zouaoui et al. (2016)
	HEK293	MTT	Zhang et al. (2014)
	Caco-2	MTT	Assunção et al. (2019)
	H9c2	MTT	Zhang et al. (2021)
	HCT116	MTT	Kwon et al. (2012)
	SK	MTT	Hanelt et al. (1994)
Aflatoxins			
AFB1, AFM1	BME	CCK-8	Wu et al. (2021)
AFB1, AFM1	Caco-2, Hep-G2, SK-N-SH	MTT, LDH release	Zheng et al. (2018)
AFM1	Caco-2	MTT	Gao et al. (2016)
AFB1	BRL 3A	MTT, LDH release	Sun et al. (2015)
AFB1	Caco-2, Raw264.7 MDBK	MTT, NR	Clarke et al. (2014)
AFB1	BME-UV1	Cell Proliferation Reagent WST-1	Ghadiri et al. (2019)
AFB1	BME-UV1	MTT, NR	Caruso et al. (2009)
AFB1, AFM1	Caco-2	MTT	J. Zhang et al. (2015)
AFB1	PK-15	MTT, LDH release	Lei et al. (2013)
AFB1	MAC-T	BrdU kit	Park et al. (2019)
AFB1	BF-2	high content screening	Zhou et al. (2017b)
AFB1	HepG-2, BEAS-2B	Cell Proliferation Reagent WST-1	McKean et al. (2006)
AFB1	SK, MDCK, HeLa	MTT	Hanelt et al. (1994)
AFB1, AFB2<br>AFB1	RTGiLL-W1	AlamarBlue, CFDA-AM, NR	Bernal-Algaba et al. (2021)
AFB1	HepG-2 Raw 264.7	AlamarBlue	Zhou et al. (2017a)
	HepG-2	high content assay	Li et al. (2018)
AFB1	primary hepatocytes of	MTT, LDH release	He et al. (2010)

	Cyprinus carpio		
OCHRATOXIN A	Caco-2	MTT	Gao et al. (2016)
	HK-2, HepG-2	MTT	Pinhão et al. (2020)
	Het-1A	CCK-8	Zhao et al. (2021)
	Caco-2, Raw264.7, MDBK	MTT	Clarke et al. (2014)
	BRL	CCK-8	Wang et al. (2020)
	MAC-T	BrdU Kit	Lee et al. (2019)
	HepG-2	MTT	Wang et al. (2014)
	Caco-2	MTT	Assunção et al. (2019)
	PBM	MTT	Stoev et al. (2009)
	RTH-149, PLHC-1, H4IIE	AlamarBlue, CFDA-AM, NR	García-Herranz et al. (2019)
	K562, PBL, PBG	MTT	Reubel et al. (1987)
	RTGiLL-W1	AlamarBlue, CFDA-AM, NR	Bernal-Algaba et al. (2021)
	HepG-2	MTT	N. Zheng et al. (2018)
	RPTC, HKC, PKC, LLC-PK1	MTT, NR	Dietrich et al. (2001)
	HEK 293	MTT	Gong et al. (2019)

	PK15	MTT	Šegvić Klarić et al. (2014)
	HepG-2	MTT	Gayathri et al. (2015)
	Vero	MTT	Bouslimi et al. (2008)
	IHKE	CCK-8 CASY cell counter	Knecht et al. (2005)
Fumonisins			
FB1	BRL 3A	MTT, LDH release	Sun et al. (2015)
FB1	HK-2, HepG-2	MTT	Pinhão et al. (2020)
FB1	CHO-K1, V79, C5-O, Caco-2, HepG-2	MTT	Cetin and Bullerman (2005)
	Caco-2, Raw264.7, MDBK	MTT, NR	Clarke et al. (2014)
FB1	PK-15	MTT, LDH release	Lei et al. (2013)
FB1	GES-1	CCK-8, LDH release	Yu et al. (2020)
FB1	IPEC-J2	MTT	Wan et al. (2013)
FB1<br>FB1	HepG-2, BEAS-2B	Cell Proliferation Reagent WST-1	McKean et al. (2006)
FB1	PBM	MTT	Stoev et al. (2009)
FB1, FB2	SK	MTT	Hanelt et al. (1994)
FB1, FB2<br>FB1	RTGiLL-W1	AlamarBlue, CFDA-AM, NRU	Bernal-Algaba et al. (2021)
DEOXYNIVALENOL	Porcine lymphocytes	CCK-8	Kachlek et al. (2017)
	HepG-2	MTT	Fernández-Blanco et al. (2018)
	BRL 3A	MTT, LDH release	Sun et al. (2015)
	Caco-2	MTT, NR	Kouadio et al. (2005)
	IPEC-12	NR	Broekaert et al. (2016)
	Jurkat T	MTT	Aupanun et al. (2019)
	CHO-K1, V79, C5-O,	MTT	Cetin and Bullerman (2005)

	Caco-2, HepG-2		
	PK-15	MTT, LDH release	Lei et al. (2013)
T-2, HT-2 toxins	IPEC-J2	MTT	Wan et al. (2013)
	BF-2 cells	high content screening	Zhou et al. (2017b)
	Vero	MTT, NR	Ruiz et al. (2011)
	RTH-149, PLHC-1, H4IIE	AlamarBlue, CFDA-AM, NR	García-Herranz et al. (2019)
	K562, PBL, PBG	MTT	Reubel et al. (1987)
	SK, MDCK, HeLa	MTT	Hanelt et al. (1994)
	RTGiLL-W1	AlamarBlue, CFDA-AM, NR	Bernal-Algaba et al. (2021)
	RTgill-W1, IPEC-1, IPEC-2, HepG-2	NR, SRB and WST-1	Mayer et al. (2017)
	SK, VERO, MDCK, BEL	MTT	Reubel G.H. (1989)
	HepG-2 and RAW 264.7	AlamarBlue	Zhou et al. (2017a)
	HepG-2	high content assay	Li et al. (2018)
	Caco-2	MTT, NR	Alassane-Kpembi et al. (2013)
	Porcine lymphocytes	CCK-8	Kachlek et al. (2017)
	primary hepatocytes of Cyprinus carpio	MTT, LDH release	He et al. (2010)
	IPEC-1	MTT	Alassane-Kpembi et al. (2015)
	HepG-2, MRC-5	AlamarBlue, BrdU	Ivanova et al. (2006)
	Leydig cells	CCK-8	Ling et al. (2020)
	HepG-2	MTT	Taroncher et al. (2020)
	SerW3	MTT, LDH release	Karacaoğlu and Selmanoğlu (2017)
	TM3 Leydig cells	MTT, LDH release	Yuan et al. (2016)
	Vero	MTT, NR	Bouaziz et al. (2006)
	HepG-2	MTT	Fernández-Blanco et al. (2018)
Zearalenone	RPTCE, NHLF	Caspase-3 activity, Hoechst 33258 dye staining	Königs et al. (2009)
	TM4	CCK-8	Yang et al. (2021)
	Veto	MTT, NR	Ruiz et al. (2011)
	SK, MDCK, HeLa	MTT	Hanelt et al. (1994)
	HepG-2, MRC-5	AlamarBlue, BrdU	Ivanova et al. (2006)
	Caco-2	MTT	Gao et al. (2016)
	BRL 3A	MTT, LDH release	Sun et al. (2015)
	Caco-2	MTT, NR	Kouadio et al. (2005)
	Cheng liver cells	MTT	Lee et al. (2013)
	CHO-K1	MTT	Tatay et al. (2014)
	CHO-K1	MTT, NR	Ferrer et al. (2009)
	CHO-K1, V79, C5-O, Caco-2, HepG-2	MTT	Cetin and Bullerman (2005)
	PK-15	MTT, LDH release	Lei et al. (2013)
	HepG-2	MTT	Wang et al. (2014)

	IPEC-J2	MTT	Wan et al. (2013)
	BF-2	high content screening	Zhou et al. (2017b)
	HepG-2	NR	Marin et al. (2019)
	K562, PBL, PBG	MTT	Reubel et al. (1987)
	SK, MDCK, HeLa	MTT	Hanelt et al. (1994)
	RTGill-W1	AlamarBlue, CFDA-AM, NR	Bernal-Algaba et al. (2021)
	SK, Vero, MDCK, BEL	MTT	Reubel G.H. (1989)
	HepG-2	MTT	N. Zheng et al. (2018)
	HepG-2, RAW 264.7	AlamarBlue	Zhou et al. (2017a)
	HepG-2	high content assay	Li et al. (2018)
	porcine lymphocytes	CCK-8	Kachlek et al. (2017)

	RTL-W1, RTGill-W1, SHK-1, RT-EQ clone 8, CCB	MTT, NR	Pietsch et al. (2014)
Citrinin			
	SK	MTT	Hanelt et al. (1994)
	PMB	MTT	Stoev et al. (2009)
	HEK 293	MTT	Gong et al. (2019)
	PK15	MTT	Šegvić Klarić et al. (2014)
	MDBK, PFBK	Nuclei counted via hemocotometer	Yoneyama et al. (1986)
	HepG-2	MTT	Gayathri et al. (2015)
	Vero	MTT	Bouslimi et al. (2008)
	HL-60	MTT, trypan blue exclusion	(Yu et al., 2006)
	human osteoblasts	MTT	Huang et al. (2009)
	IHKE	CCK-8, CASY assay	Knecht et al. (2005)
Enniatins	A549	AlamarBlue	Johannessen et al. (2007)
ENA, ENA1, ENB, ENB1			
	Caco-2	MTT,	Prosperini et al. (2014)
ENA, ENA1, ENB, ENB1	RTGill-W1	AlamarBlue, CFDA-AM, NR	Bernal-Algaba et al. (2021)
ENA, ENA1, ENB, ENB1	IPEC-J2	Flow cytometry	Fraeyman et al. (2018)
ENA, ENA1, ENB, ENB1	HepG-2, MRC-5	AlamarBlue, BrdU	Ivanova et al. (2006)
ENA, ENA1, ENB, ENB1	Caco-2	MTT	Meca et al. (2010)

ENA, ENA1, ENA2, ENB, ENB1, ENB4, ENJ3	Caco-2, HepG-2, HT29	MTT	Meca et al. (2011)
ENB	Jurkat-T	MTT	Manyes et al. (2018)
ENA, ENA1, ENB, ENB1	CHO-K1	MTT	Lu et al. (2013)
ENB	RAW 267.4	AlamarBlue, NR	Gammelsrud et al. (2012)
Combinations of 2 mycotoxins			

DON, PAT, T-2	HepG-2	MTT	Fernández-Blanco et al. (2018)
AFM1, OTA, ZEN	Caco-2	MTT	Gao et al. (2016)
DON, ZEN, AFB1	BRL 3A	MTT, LDH release	Sun et al. (2015)
OTA, FB1	HK-2, HepG-2	MTT	Pinhão et al. (2020)
T-2, HT-2	Leydig cells	CCK-8	Ling et al. (2020)
AFB1, FB1, OTA	MDBK	MTT, NR	Clarke et al. (2014)
OTA, ZEN	HepG-2	MTT	Wang et al. (2014)
OTA, PAT	Caco-2	MTT	Assunção et al. (2019)
DON, FB1, ZEN	IPEC-J2	MTT	Wan et al. (2013)
ENA, ENA1, ENB, ENB1	Caco-2	MTT	Prosperini et al. (2014)
ZEN, DON, AFB1	BF-2	high content screening	Zhou et al. (2017b)
AFB1, FB1	HepG-2, BEAS-2B	Cell Proliferation Reagent WST-1	McKean et al. (2006)
DON, T-2 OTA, ZEN	Vero	NR	Ruiz et al. (2011)
	HepG-2	MTT	N. Zheng et al. (2018)
AFB1, ZEN, DON	HepG-2, RAW 264.7	AlamarBlue	Zhou et al. (2017a)
ZEN, DON, AFB1	HepG-2	high content assay	Li et al. (2018)
ZEN, DON, FB1	porcine lymphocytes	CCK-8	Kachlek et al. (2017)
DON, AFB1	primary hepatocytes	MTT, LDH release	He et al. (2010)
OTA, CIT	HEK 293	MTT	Gong et al. (2019)
OTA, CIT	PK15	MTT	Šegvić Klarić et al. (2014)
OTA, CIT	HepG-2	MTT	Gayathri et al. (2015)
OTA, CIT	Vero	MTT	Bouslimi et al. (2008)
ENA, ENA1, ENB,	CHO-K1	MTT	(Lu et al., 2013)
ENB1<br>CIT, OTA	IHKE	Caspase-3 activity	Knecht et al. (2005)
Combinations of 3 mycotoxins			
PAT, DON, T-2	HepG-2	MTT	Fernández-Blanco et al. (2018)
AFM1, OTA, ZEN	Caco-2	MTT	Gao et al. (2016)
OTA, AFB1, FB1<br>DON, FB1, ZEN	caco-2, RAW264.7, MDBK	MTT, NR	Clarke et al. (2014)
ENA, ENA1, ENB, ENB1	IPEC-J2	MTT	Wan et al. (2013)
ZEN, DON, AFB1	Caco-2	MTT	Prosperini et al. (2014)
OTA, FB1, CIT	BF-2	high content screening	Zhou et al. (2017b)
AFB1, ZEN, DON	PBM	MTT	Stoev et al. (2009)
ZEN, DON, AFB1 DON,	HepG-2, RAW 264.7	AlamarBlue	Zhou et al. (2017a)
ZEN, FB1	HepG-2	high content assay	Li et al. (2018)
ENA, ENA1, ENB,	porcine lymphocytes	CCK-8	Kachlek et al. (2017)
ENB1	CHO-K1	MTT	Lu et al. (2013)
Combinations of 4 mycotoxins			
ENA, ENA, ENB, ENB1	Caco-2	MTT	Prosperini et al. (2014)
